# Rethinking Mediterranean Diet Adherence and Body Composition: A 3-Year Prospective Observational Study in Patients with Type 2 Diabetes and Arterial Hypertension

**DOI:** 10.3390/nu18152482

**Published:** 2026-08-01

**Authors:** Dora Bučan Nenadić, Josipa Radić, Ela Kolak Gaurina, Marija Selak, Mislav Radić

**Affiliations:** 1 Department of Nutrition and Dietetics, University Hospital of Split, 21000 Split, Croatia; dora.bucan@kbsplit.hr (D.B.N.); ela.kolak@kbsplit.hr (E.K.G.); marija.selak@kbsplit.hr (M.S.); 2 Department of Internal Medicine, School of Medicine, University of Split, 21000 Split, Croatia; mislav.radic@kbsplit.hr; 3 Department of Internal Medicine, Division of Nephrology and Haemodialysis, University Hospital of Split, 21000 Split, Croatia; 4 Department of Internal Medicine, Division of Rheumatology, Allergology and Clinical Immunology, University Hospital of Split, 21000 Split, Croatia

**Keywords:** type 2 diabetes mellitus, arterial hypertension, Mediterranean diet, body composition, dietitian follow-up, cardiometabolic disease, chronic kidney disease

## Abstract

**Background/Objectives**: Type 2 diabetes mellitus (T2DM) and arterial hypertension (AH) frequently coexist and are major risk factors for cardiovascular disease (CVD) and chronic kidney disease. Although the Mediterranean diet (MeDi) is widely recommended in cardiometabolic management, longitudinal real-world evidence regarding dietary adherence, body composition, and the role of continuous dietitian-led follow-up remains limited. **Methods**: This three-year prospective observational study included 158 adults with T2DM and AH treated at the Outpatient Clinic for Clinical Nutrition, University Hospital Centre Split, Croatia. Body composition was assessed using multi-frequency bioelectrical impedance analysis, dietary adherence using the Mediterranean Diet Serving Score (MDSS), and renal and metabolic parameters using standard laboratory methods. Participants were stratified according to attendance at scheduled dietitian-led follow-up visits. **Results**: Significant reductions were observed in body weight, body mass index (BMI), waist circumference, muscle mass, skeletal muscle index, and phase angle (PhA) during follow-up (all *p* < 0.001). Lipid profile improved, with reductions in total cholesterol and low-density lipoprotein levels and an increase in high-density lipoprotein concentrations. In contrast, renal function declined, as evidenced by increased creatinine and urea levels and decreased estimated glomerular filtration rate (eGFR). CVD incidence increased significantly during follow-up (*p* < 0.001). Although overall adherence to the MeDi improved, only 17.8% of participants achieved high adherence. Participants attending regular dietitian-led follow-up demonstrated significantly higher MDSS scores, greater adherence to the MeDi, and higher phase angle values compared with participants without regular follow-up. **Conclusions**: Adults with T2DM and AH demonstrated partial enhancement of MeDi adherence over three years; although the lipid profile also improved, this could not be attributed to diet and may largely reflect pharmacological treatment. These changes were accompanied by progressive muscle mass loss, declining renal function, and increased cardiovascular burden. Continuous dietitian-led follow-up was associated with superior dietary quality and better nutritional status, supporting the importance of individualized, multidisciplinary approaches integrating sustained nutritional counselling, lean mass preservation, and structured physical activity in long-term cardiometabolic care.

## 1. Introduction

Type 2 diabetes mellitus (T2DM) and arterial hypertension (AH) frequently coexist and represent leading risk factors for cardiovascular disease (CVD) and chronic kidney disease (CKD) [1]. Comorbidities associated with T2DM and AH accelerate the progression of renal dysfunction and adversely affect body composition characterized by increased adiposity and loss of skeletal muscle mass, thereby further increasing morbidity and mortality in this population [1].

Over recent decades, growing attention has been devoted to dietary patterns as modifiable risk factors, with the Mediterranean diet (MeDi) emerging as a central focus. Regarding renal function, an increasing body of evidence points toward a protective effect of the MeDi in individuals with metabolic disorders [2]. Recent studies and narrative reviews suggest that adherence to the MeDi is associated with a lower incidence and slower progression of diabetic kidney disease [3], including more favorable changes in estimated glomerular filtration rate (eGFR) and albuminuria among populations with T2DM and obesity [4]. Although interventional studies remain limited, a growing number of publications are drawing attention to the potential role of the MeDi in preserving renal function [3,5].

Beyond its effects on clinical outcomes, the MeDi has also been investigated with respect to body composition [6]. Meta-analyses and recent clinical trials indicate that adherence to the MeDi, particularly when combined with tailored energy restriction and/or structured physical activity, can lead to reductions in body weight and waist circumference, as well as favorable shifts in fat mass and lean body mass [6,7]. However, effects on skeletal muscle mass and overall body composition quality remain heterogeneous across studies, underscoring the importance of individualized approaches and ongoing monitoring [8].

The clinical benefits of established dietary patterns are contingent upon sustained adherence, with structured nutritional counselling and regular follow-up serving as critical determinants of success. Interventions incorporating individualized dietary counselling delivered in person or supported by digital tools have consistently demonstrated superior uptake and long-term adherence to recommended dietary modifications, thereby enhancing their clinical effectiveness [9]. However, despite this evidence, longitudinal data examining the impact of regular nutritional follow-up in real-world settings remain scarce, particularly among high-risk populations with T2DM and AH [9,10].

Specifically, few studies have evaluated whether ongoing dietitian-led follow-up under routine clinical conditions translates into improved adherence to the MeDi, and whether such adherence is associated with favorable changes in body composition and preservation of renal function over time [11].

Given the well-established benefits of the MeDi, the poor findings of our baseline study—namely, the high prevalence of overweight and obesity (88.3%) and the very low overall adherence to the MeDi (8.9%)—provided the rationale for the present study [12,13]. The aim of this three-year prospective observational study was to evaluate adherence to the Mediterranean diet (MeDi) and changes in body composition parameters over a three-year follow-up period in individuals with T2DM and AH managed in real-world clinical practice.

## 2. Materials and Methods

### 2.1. Study Population

The study population initially included 248 participants who were recruited for a baseline analysis. All participants were adults with T2DM and AH (≥18 years) and provided written informed consent [12].

**Figure 1:**
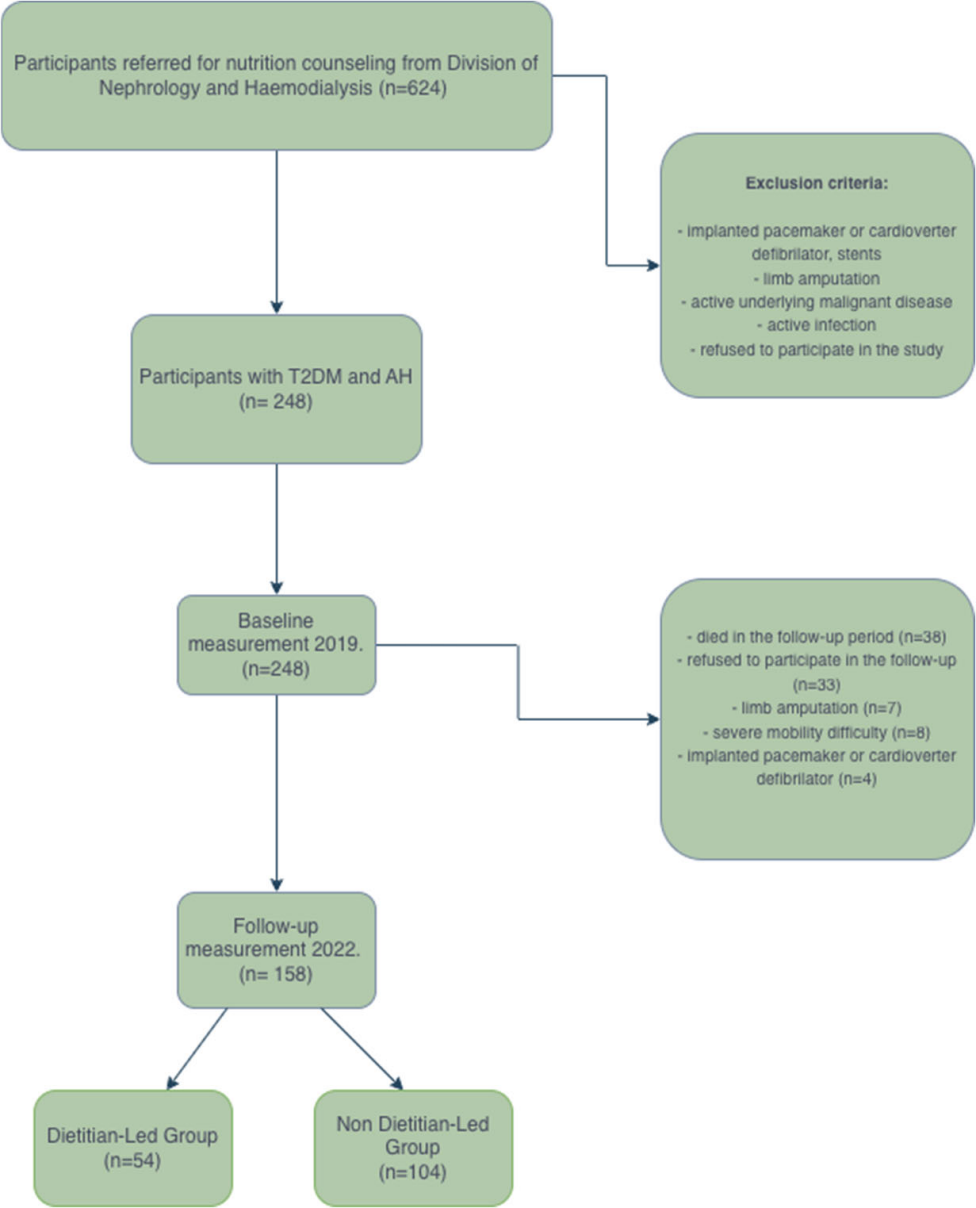
The study flow-chart. Abbreviations: n—number; T2DM—type 2 diabetes mellitus; AH—arterial hypertension.

Three years after the initial assessment, all participants were re-invited to attend a follow-up evaluation. Of the original cohort, 158 participants were included in the final analysis, while 90 were lost to follow-up, including 38 who died during the follow-up period, 33 who declined to participate, 7 who underwent limb amputation, 8 who developed severe mobility limitations, and 4 who had a pacemaker or cardioverter-defibrillator implanted during the follow-up period. The study flowchart illustrating attrition from the initial 248 to the final 158 analyzed participants is presented in Figure 1.

Following the initial visit, at which each participant received individualized nutritional education based on their medical history and grounded in the principles of the MeDi, with emphasis on the dietary management of T2DM and AH, furthermore supported by visual materials and individual dietary plan, follow-up consultations with the same dietitian were scheduled every two months during the first two years and every four months during the third year, amounting to 15 scheduled follow-up visits over the three-year period. Participants were classified as regularly followed (*n* = 54) if they continued to attend the scheduled follow-up visits beyond the initial period, and as not regularly followed (*n* = 104) if they did not return after the initial visit or attended only the first follow-up visit and received no subsequent nutritional monitoring.

All participants were examined at the baseline and follow-up at an Outpatient Clinic for Clinical Nutrition, Department of Internal Medicine, Division of Nephrology and Haemodialysis, University Hospital Centre Split, Croatia, with comprehensive assessments of body composition, laboratory parameters, and dietary adherence.

### 2.2. Follow-Up Assessments

#### 2.2.1. Body Composition and Anthropometric Parameters

Body composition was assessed using a multi-frequency bioelectrical impedance analysis (BIA) device MC-780 Multi Frequency Segmental Body Mass Analyzer (Tanita, Tokyo, Japan) [14]. Measurements were obtained at three frequencies (5, 50, and 250 kHz) with a measurement current of ≤90 µA, using a tetrapolar segmental configuration (both feet on stainless-steel electrodes and both hands on the plated handgrip electrodes). Parameters measured included fat mass (kg and %), fat-free mass (kg), trunk fat mass (kg and %), muscle mass (kg), skeletal muscle index (SMI; kg/m^2^), and phase angle (PhA; °). Fat-free mass, muscle mass, and skeletal muscle index were derived using the manufacturer’s proprietary prediction algorithms, which are not publicly disclosed. Phase angle was calculated directly from the measured resistance and reactance at 50 kHz (phase angle = arctangent [reactance/resistance] × 180°/π). Participants were instructed to avoid food and fluid intake for at least three hours prior to measurement, and to avoid alcohol, extreme physical activity, or large meals the day before assessment. Height, weight, waist circumference (WC), hip circumference (HC) and mid-upper arm circumference (MUAC) were measured using standard stadiometers and flexible, non-stretchable measuring tapes [15]. Body mass index (BMI) was calculated for each participant.

#### 2.2.2. Renal Function and Metabolic Parameters

Laboratory parameters collected included fasting blood glucose (FBG), glycated hemoglobin (HbA_1_C), serum creatinine, urea, uric acid, albumin, electrolytes (sodium, potassium, phosphate, calcium), C-reactive protein (CRP), lipid profile (total cholesterol, low-density lipoprotein cholesterol (LDL), high-density lipoprotein cholesterol (HDL) and triglycerides), and complete blood count. Urinary albumin was assessed from 24 h urine collections. Estimated glomerular filtration rate (eGFR) was calculated using the CKD-EPI equation. Blood samples and urine collections were performed on the same day as the follow-up body composition assessment. Blood samples for analysis of serum levels were collected in standard test tubes without additives in our Laboratory of Medical Diagnostics and Biochemistry at the University Hospital of Split, Croatia, and 30 min later, were centrifuged for 10 min at 1690× *g* on HERMLE Z400 centrifuge model (Hermle Labortechnik GmbH, Wehingen, Germany). For creatinine measurement, the Jaffe method was used. A complete blood count was obtained using a hematology analyzer (Advia 120, Siemens, Erlangen, Germany).

#### 2.2.3. Mediterranean Diet Serving Score

Adherence to the MeDi was assessed using the Mediterranean Diet Serving Score (MDSS) questionnaire [16] validated in the Croatian population [17]. Participants reported the frequency of consumption of 14 food groups. The MDSS categorizes dietary intake into fourteen food groups, each assigned a specific point value based on the revised Mediterranean food pyramid. Fruits, vegetables, olive oil, and cereals receive up to three points when consumed at every meal, while dairy products and nuts contribute two points upon meeting daily intake recommendations. The remaining food groups—potatoes, legumes, eggs, fish, white meat, red meat, sweets, and fermented beverages—each yield one point when consumed within their respective recommended weekly frequencies. Scores were calculated according to the revised Mediterranean food pyramid, with higher scores indicating greater adherence. A cut-off score of ≥13.5 defined adherence to the MeDi [16,18].

#### 2.2.4. Additional Data Collection

All clinical events occurring over the three-year follow-up period—including CVD and cerebrovascular disease, hospitalizations, surgeries, COVID-19 infection, and mortality, as well as the events accounting for attrition (deaths, limb amputations, severe mobility limitations, and pacemaker/cardioverter-defibrillator implantation)—were ascertained retrospectively at the follow-up assessment and covered the entire interval between baseline and follow-up, rather than a single time point. Data were obtained from participants’ hospital and clinical medical records and, where applicable, supplemented by participant report; wherever possible, self-reported events were verified against the medical documentation. CVD was defined as any documented diagnosis of ischaemic heart disease, heart failure, or peripheral arterial disease recorded in the participants’ medical records. Cerebrovascular disease was recorded and reported as a separate variable and was not included in the CVD category. Arrhythmias, revascularisation procedures, and venous thromboembolic disease were not classified as CVD in this study. Hospitalizations were defined as prolonged inpatient admissions of at least seven days, selected for their clinical relevance to skeletal muscle mass given the association between prolonged immobilization and muscle loss; surgeries were defined as major procedures (cardiac, abdominal, vascular, urological), with minor procedures (e.g., excision of skin lesions or dental procedures) excluded; COVID-19 infection was based on a documented positive SARS-CoV-2 test or participant report; and mortality was recorded as all-cause death. Disease progression was assessed from available medical data, including patient history, laboratory parameters, and the medical classification of disease and was not based on a single predefined, standardized index combining these parameters. These events were compiled for descriptive purposes and are reported as cumulative frequencies over the follow-up period; they were not modelled as time-to-event outcomes and were not entered as covariates in the between-group comparisons, which should be considered when interpreting the findings.

Systematic data on pharmacological therapy were not available. Because no unified hospital information system existed at the time of the baseline assessment, baseline medication data could not be reliably retrieved, and consequently changes in medication type, dose, or the initiation of new treatments over the follow-up period could not be determined. Physical activity was initially recorded without a validated instrument and was therefore removed from the analysis.

### 2.3. Statistical Analysis

Categorical data are presented as absolute and relative frequencies. Differences in categorical variables between measurements were tested using the McNemar–Bowker test or the Marginal Homogeneity Test. The normality of distribution of continuous variables was assessed using the Shapiro–Wilk test. Continuous data are presented as medians with interquartile ranges. Differences in continuous variables between two independent groups were tested using the Mann–Whitney U test, while differences between two measurement points were tested using the Wilcoxon signed-rank test. All *p* values were two-sided. The significance level was set at α (alpha) = 0.05. Statistical analysis was performed using MedCalc^®^ Statistical Software version 23.5.2 (MedCalc Software Ltd., Ostend, Belgium; https://www.medcalc.org; 2026).

## 3. Results

A total of 248 adult participants (≥18 years) with T2DM and AH were initially screened for baseline assessment. After three years, 158 participants remained for present study, while 90 were lost to follow-up due to 38 deaths, 33 withdrawals, and 19 due to different complications as detailed in the study flowchart. To assess the potential for attrition bias, baseline characteristics of participants who completed the 3-year follow-up (*n* = 158) were compared with those lost to follow-up (*n* = 90). As shown in Table 1, several significant differences were observed between the groups. Participants lost to follow-up were significantly older than those who completed the study (71 [65–77] vs. 65 [58–72] years, *p* < 0.001). They also had a higher prevalence of CVD (18.5% vs. 7.6%, *p* = 0.02). Differences were also seen in meal frequency distribution (*p* = 0.02), with a greater proportion of participants lost to follow-up reporting only one to two meals per day. Regarding laboratory parameters, participants lost to follow-up exhibited significantly poorer renal function at baseline, including higher serum urea (11.9 [8.4–16.5] vs. 8.2 [6.0–12.2], *p* < 0.001), higher creatinine level (148 [94.5–205.5] vs. 112.5 [76–155], *p* = 0.001), greater proteinuria (879 [195–2466] vs. 338.5 [93.5–1095], *p* = 0.02), and lower eGFR (35.5 [23.9–69.0] vs. 55.4 [35.3–80.3], *p* < 0.001).

A comparison of patient characteristics at baseline and follow-up is presented in Table 2, demonstrating significant changes in BMI (*p* = 0.02) and meal frequency (*p* < 0.001). During the 3-year follow-up period, participants underwent monitoring for key clinical outcomes (Table 2). A statistically significant increase in CVD prevalence was observed (*p* < 0.001), whereas CKD prevalence increased by 8.9%; however, this change was not statistically significant. Notable event rates included surgical interventions in 31 participants (19.6%), hospitalizations in 64 (40.5%), and COVID-19 infections in 83 (52.5%). These figures are reported descriptively to characterize the clinical burden experienced by this high-risk cohort during follow-up and were not analyzed as study outcomes in relation to body composition, renal function, or nutritional status.

**Figure 2:**
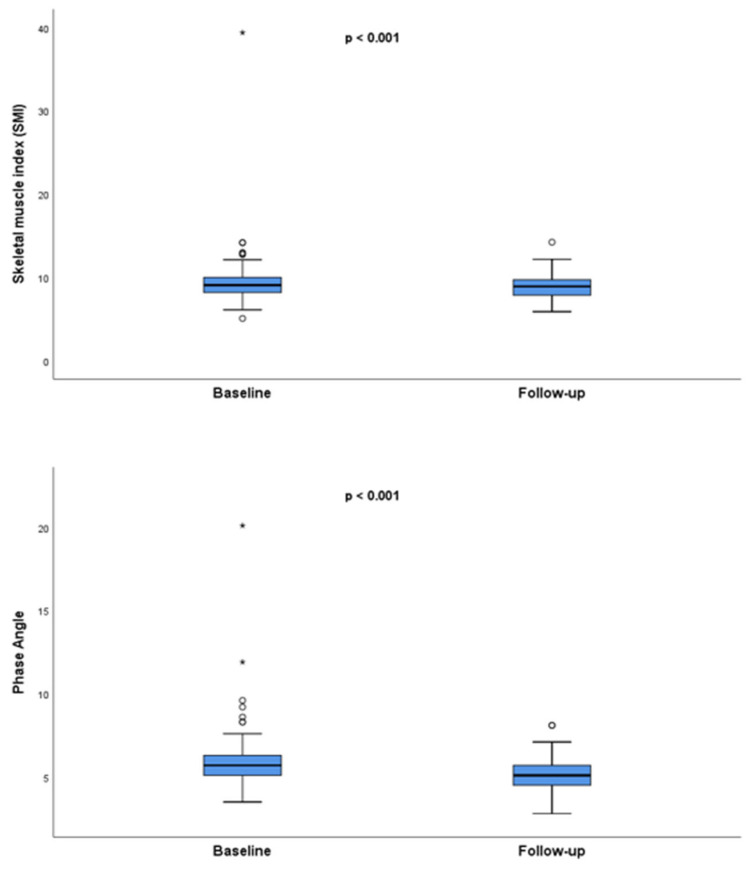
Changes in skeletal muscle index and phase angle in the follow-up period among the study population. Only statistically significant parameters are shown. Circles denote outliers; Asterisks denote extreme outliers.

Comparison of body composition and anthropometric parameters from baseline to follow-up, as presented in Supplementary Table S1 and Figure 2 (only statistically significant parameters are shown), revealed significant changes in most of the body composition parameters. Significant decreases in total weight [95.15 (IQR 81.58–108.63) vs. 92 (IQR 79.1–104.43); *p* < 0.001], BMI [30.9 (IQR 27.83–34.88) vs. 29.9 (IQR 27–33.33); *p* < 0.001], MUAC [32.6 (IQR 29.98–36) vs. 30 (IQR 28–33.5); *p* < 0.001], WC [111 (IQR 102.53–120) vs. 104 (IQR 96–116); *p* < 0.001], and HC [113.5 (IQR 106.38–124.13) vs. 108.25 (IQR 101–118.5); *p* < 0.001] were observed. In terms of body composition, significant reductions in body fat [29 (IQR 23.25–36.68) vs. 27.35 (IQR 19.35–34.6); *p* = 0.01], fat-free mass [65.85 (IQR 58.78–75.03) vs. 63.9 (IQR 57.1–72.38); *p* < 0.001], muscle mass [62.55 (IQR 55.78–71.23) vs. 60.7 (IQR 54.2–68.78); *p* < 0.001], SMI [9.08 (IQR 8.19–10) vs. 8.91 (IQR 7.85–9.74); *p* < 0.001], and PhA [5.7 (IQR 5.08–6.3) vs. 5.1 (IQR 4.5–5.7); *p* < 0.001] were observed, whereas significant increase in trunk fat mass [28.5 (IQR 22.35–34.03) vs. 29.8 (IQR 22.65–34.6); *p* = 0.02] was observed after three years.

**Figure 3:**
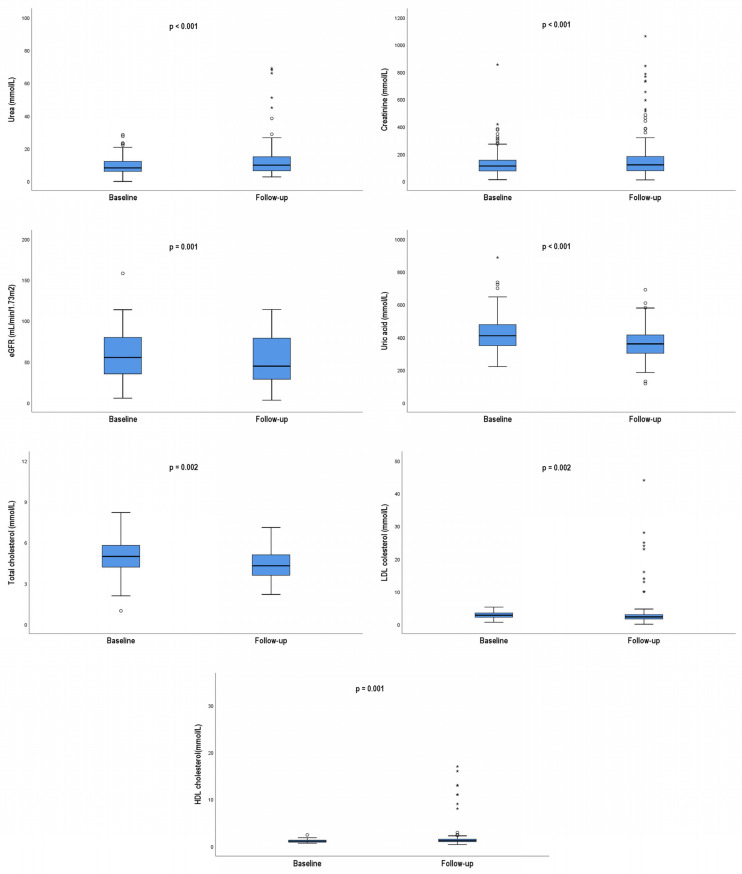
Changes in the laboratory parameters in the follow-up period among the study population. Abbreviations: eGFR—estimated glomerular filtration rate; LDL—low-density lipoprotein; HDL—high-density lipoprotein. Only statistically significant parameters are shown. Circles denote outliers; Asterisks denote extreme outliers.

Figure 3 (only statistically significant parameters are shown) and Supplementary Table S2 display the differences in biochemical parameters between the two measurements. Statistically significant increases in urea [8.2 (IQR 6–12.2) vs. 9.9 (IQR 6.4–15.1); *p* < 0.001], creatinine [112.5 (IQR 76–155) vs. 120.5 (IQR 77.75–184.5); *p* < 0.001], and HDL level [1.1 (IQR 0.9–1.3) vs. 1.2 (IQR 1–1.5); *p* = 0.001] were found. Conversely, a significant decrease in eGFR [55.4 (IQR 35.3–80.28) vs. 44.7 (IQR 28.9–78.9); *p* = 0.001], total cholesterol [4.99 (IQR 4.2–5.8) vs. 4.3 (IQR 3.55–5.15); *p* = 0.002], LDL [2.8 (IQR 2.2 to 3.5) vs. 2.3 (IQR 1.7–3); *p* = 0.002], and uric acid level [409 (IQR 348–477) vs. 360 (IQR 303–414); *p* < 0.001] was found.

**Figure 4:**
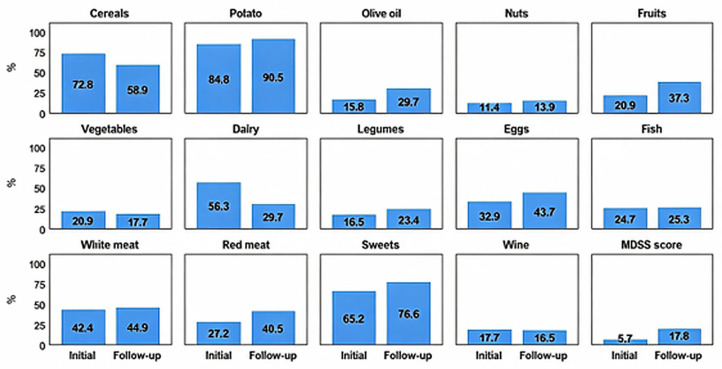
Percentage of participants meeting the Mediterranean Diet Serving Score (MDSS) criterion for each dietary component at baseline and follow-up. Values represent the proportion of participants fulfilling the recommended MDSS criterion for each food category (not the actual dietary intake). Higher percentages indicate greater adherence to the MDSS recommendation. The denominator for all percentages was the total study population (*n* = 158).

Figure 4 presents changes in adherence to the MDSS recommendations between baseline and follow-up. A significant increase in the overall MeDi adherence was observed at follow-up; when applying the originally proposed cut-off (≥13.5), 17.8% of participants were classified as adherent. The proportion of participants meeting the MDSS recommendation significantly decreased consumption of cereals [115 (72.8%) vs. 93 (58.9%); *p* = 0.008], red meat [43 (27.2%) vs. 64 (40.5%)], dairy [89 (56.3%) vs. 47 (29.7%); *p* < 0.001], and sweets [103 (65.2%) vs. 121 (76.6%); *p* = 0.01], as well as a significant increase in the intake of olive oil [25 (15.8%) vs. 47 (29.7%); *p* = 0.003], fruits [33 (20.9%) vs. 59 (37.3%); *p* = 0.002], and eggs [52 (32.9%) vs. 69 (43.7%); *p* = 0.03].

For the further analysis, all participants were divided into two groups: patients regularly followed by a dietitian and those not followed by a dietitian. At baseline, all participants received individualized dietary education from a dietitian, based on participants’ medical history and grounded in the principles of the MeDi with emphasis on dietary management of T2DM and AH. Scheduled follow-up consultations with the same dietitian were implemented to monitor adherence and modify dietary recommendations as necessary. After the three-year follow-up period, 54 of the 158 participants (34.2%) consistently attended dietitian-led monitoring sessions, whereas the remaining 104 participants did not maintain regular attendance.

**Figure 5:**
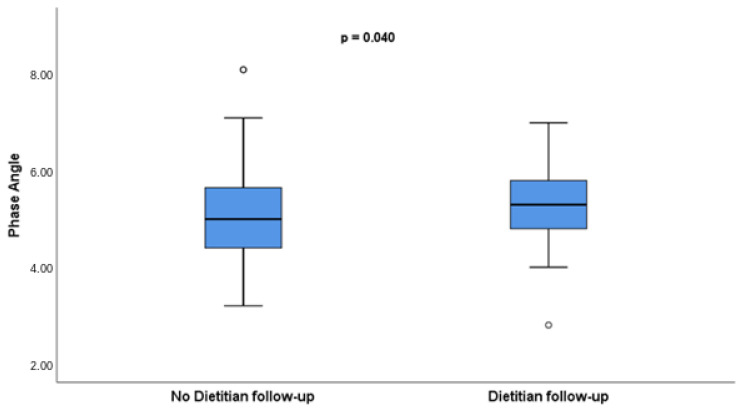
Differences in phase angle among study participants according to the dietitian follow-up. Circles denote outliers.

As shown in Supplementary Table S3 and Figure 5 (only statistically significant parameters are shown), no significant differences were observed in body composition and anthropometric parameters between the two subgroups, except for a higher phase angle [5.0 (IQR 4.4–5.68) vs. 5.3 (IQR 4.8–5.8); *p* = 0.04] in patients followed by a dietitian.

**Figure 6:**
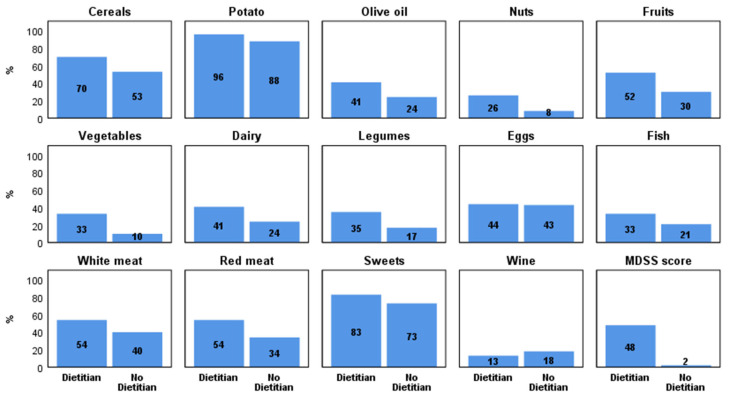
Percentage of participants meeting the Mediterranean Diet Serving Score (MDSS) recommendation for each dietary component according to dietitian follow-up. Values represent the proportion of participants fulfilling the recommended MDSS criterion for each food category. Higher percentages indicate greater adherence to the respective recommendations.

Figure 6 presents differences in adherence to the MeDi recommendations according to dietitian follow-up. Patients followed by a dietitian demonstrated a significantly higher MDSS [7 (IQR 5–9) vs. 12 (IQR 8–14); *p* < 0.001]. Within this subgroup, when applying the originally proposed cut-off (≥13.5), 48% were classified as adherent to the MeDi, with a median total MDSS of 12. This subgroup also reported significantly higher consumption of cereals, olive oil, nuts, fruits, vegetables, dairy, and legumes, as well as lower consumption of red meat.

## 4. Discussion

The present study provides important longitudinal insights into changes in nutritional habits, anthropometric and laboratory parameters, body composition, and cardiometabolic health in patients with T2DM and AH over a three-year period. However, interpretation of the findings should consider the characteristics of participants lost to follow-up. Individuals who did not complete the follow-up were older and clinically more vulnerable at baseline, with a higher burden of CVD and more advanced renal impairment. These findings suggest that attrition was not entirely random and may have resulted in a relatively healthier cohort being retained for longitudinal analyses, potentially leading to an underestimation of disease progression and adverse outcomes over time.

Despite this potential survivor effect, our findings revealed several important trends. Although participants experienced a reduction in body weight and improvements in selected lifestyle-related parameters, these changes were accompanied by a decline in skeletal muscle mass and PhA, which indicate deteriorating nutritional and functional status. Furthermore, the increased incidence of CVD observed during follow-up highlights the complex and multifactorial nature of cardiometabolic progression in this high-risk population.

### 4.1. Changes in Anthropometric and Body Composition Parameters

A significant reduction in body weight, BMI [19] and other anthropometric indices (MUAC, WC, HC) was observed over time in the present study. Similarly, nutritional intervention from the PREDIMED-Plus trial yielded significant improvements in WC, glycemic control, triglyceride levels, and HDL after 1 year [20]. Over 3 years, it sustained weight loss and cognitive benefits; however, absent intensive resistance training, it was associated with muscle mass reduction despite overall visceral fat diminution, potentially resulting in a relative increase in trunk fat proportion relative to lean mass [20]. While weight loss is generally considered beneficial in T2DM and AH management, our results suggest that such reductions may not necessarily reflect improvements in overall metabolic health [21,22]. An important consideration is whether the observed weight loss was intentional, recommended as part of the dietary intervention, or unintentional and disease-related, a distinction of considerable clinical importance. On one hand, given that the majority of participants were overweight or obese at baseline (88.3%), and that the intervention included dietary counselling aimed at weight management, a proportion of the observed weight reduction was likely intentional. On the other hand, the concurrent decline in muscle mass, fat-free mass, SMI, and PhA, together with the increasing burden of CVD, accrual of comorbidities, and the high attrition and mortality observed during follow-up, suggests that at least part of the weight loss reflected an unintentional, catabolic process consistent with sarcopenic obesity rather than a favourable, intervention-driven change. This distinction is clinically critical, as unintentional weight loss and loss of lean mass are established poor prognostic indicators in T2DM, having been associated with increased all-cause mortality [23], whereas the prognostic impact of intentional weight loss remains less clear [24]. Because intentionality of weight change, appetite, and markers of catabolism were not systematically recorded, our study cannot reliably distinguish these two components, and the observed reductions in body weight should therefore be interpreted with caution rather than assumed to represent a uniformly beneficial outcome.

The observed pattern of declining muscle mass, fat-free mass, and SMI, alongside a relative increase in truncal fat, likely reflects a multifactorial interplay inherent to sarcopenic obesity, a condition marked by concomitant adiposity and skeletal muscle deterioration with significant cardiometabolic and functional consequences. Furthermore, changes in body composition may be associated with COVID-19 infection during the follow-up period as confirmed in several studies [25,26].

Notably, the observed decrease in fat-free mass, muscle mass, and SMI indicates that weight loss was, at least in part, driven by the loss of metabolically active muscle tissue [27]. This is clinically relevant, as preservation of muscle mass is essential for maintaining insulin sensitivity, functional capacity, and long-term metabolic stability.

The decline in phase angle further supports the presence of deteriorating cellular integrity and nutritional status [28]. PhA is increasingly recognized as an integrative marker of cell membrane function, body cell mass, and inflammation, and its reduction has been associated with adverse outcomes across various chronic diseases, including T2DM and CKD. In this context, the simultaneous reduction in muscle mass and PhA may reflect an underlying catabolic state, possibly driven by chronic low-grade inflammation, insulin resistance, and disease progression [29,30,31,32].

Previous studies involving individuals with chronic non-communicable diseases, including T2DM, have consistently linked higher PhA values with greater muscle mass, lower inflammation, and a more favorable metabolic profile [33]. Accordingly, nutritional interventions, particularly those ensuring adequate energy and protein intake, typically result in stabilization or an increase in PhA [34]. The decrease in PhA observed in this study may be explained by several mechanisms. Interpreting phase angle warrants particular caution in this population, as it is directly influenced by hydration status. Because PhA depends on the resistance and reactance of body tissues, it is sensitive to shifts in total body water between the intracellular and extracellular compartments [35,36]. In CKD, impaired sodium and water handling often causes extracellular fluid expansion, which lowers tissue resistance and may artificially reduce PhA, independent of genuine changes in cell mass [37]. Diuretic therapy, commonly used in both AH and CKD, can further introduce variability by acutely altering fluid balance. Given the high prevalence of CKD (62%) and antihypertensive treatment in our cohort, the observed decline in PhA may reflect not only deterioration in cellular integrity and muscle mass but also fluctuations in fluid status, underscoring the need to interpret it as an integrative rather than strictly nutritional marker.

Furthermore, a reduction in fat-free mass, particularly skeletal muscle mass, may have occurred during the intervention, especially with hypocaloric diets lacking sufficient protein intake or physical activity [38]. Finally, the ongoing effects of chronic inflammation and metabolic stress, frequently present in this population, may have negatively affected cellular integrity despite the nutritional intervention [39].

### 4.2. Renal and Cardiometabolic Changes

The observed increase in urea and creatinine, as well as decrease in eGFR, suggests a decline in renal function over time, which is consistent with the increased risk of CKD associated with older age, T2DM, and AH. Chronic hyperglycemia, AH, and inflammation are well-established drivers of progressive nephron damage and fibrosis. Recent evidence confirms that both T2DM and AH independently and synergistically contribute to structural and functional renal impairment, even in early stages of disease [40]. Furthermore, AH remains a key determinant of renal disease progression, with poorly controlled blood pressure accelerating decline in glomerular filtration and increasing cardiovascular morbidity [41]. Contemporary studies highlight that fluctuations in eGFR can occur due to therapeutic interventions, changes in intraglomerular pressure, and pharmacological treatments, which may initially reduce eGFR but confer long-term nephroprotection [42].

In parallel, the lipid profile improved, with reductions in total cholesterol and LDL and an increase in HDL. However, this improvement cannot be attributed to dietary change. Data on pharmacotherapy, including statin initiation and dose adjustments, were not systematically collected, and lipid-lowering treatment is the principal determinant of LDL reduction in this population. The observed lipid changes are therefore most plausibly explained, at least in part, by intensification of pharmacological therapy over the three-year period, and any dietary contribution cannot be isolated within this study design.

The coexistence of improving lipid parameters alongside worsening renal markers underscores the dissociation between traditional metabolic targets and overall disease progression [43]. This finding aligns with emerging evidence suggesting that cardiometabolic diseases should be viewed as systemic and progressive conditions, where improvements in isolated biochemical markers may not adequately capture the underlying pathophysiological burden [44].

Importantly, our findings also demonstrate a significant increase in CVD incidence during the follow-up period, despite improvements in certain lifestyle parameters such as meal frequency. This apparent paradox underscores the progressive nature of cardiometabolic disorders, where improvements in isolated risk factors may be insufficient to counterbalance the cumulative burden of disease. The coexistence of sarcopenia with cardiometabolic disease may further exacerbate cardiovascular risk through mechanisms involving reduced glucose disposal, increased adiposity, and systemic inflammation, as well as kidney function decline [45,46,47].

Another important aspect is the high attrition rate, with a substantial proportion of participants lost to follow-up due to death and complications. This finding itself reflects the high-risk nature of this population and aligns with previous studies demonstrating increased morbidity and mortality in patients with combined T2DM and AH [26,48]. It also suggests that those who remained in the study may represent a relatively healthier subset, potentially underestimating the true magnitude of adverse changes.

### 4.3. Dietary Changes and Mediterranean Diet Adherence

In the present study, a significant increase in overall adherence to the MeDi, as assessed by the MDSS, was observed over the three-year follow-up period. However, when applying the originally proposed cut-off (≥13.5), only a minority of participants (17.8%) were classified as adherent, indicating that, despite measurable improvements, optimal dietary patterns were not fully adopted. This finding is consistent with recent evidence suggesting that achieving and maintaining high adherence to the MeDi in real-world populations remains challenging due to lifestyle, cultural, and environmental factors [49]. A systematic review published in 2025 showed that in individuals with established T2DM, adherence to the MeDi is often low to moderate and is influenced by a range of sociodemographic and behavioral factors [50]. Furthermore, Martínez-González et al. noticed that repeated assessment of dietary adherence over time provides greater methodological value than a single baseline measurement, as it more accurately reflects sustained exposure to the dietary pattern [51].

The observed dietary changes, including reduced consumption of red meat and sweets and increased intake of olive oil and fruits, are in line with core MeDi principles proposed by Monteagudo et al. [16]. These modifications are clinically relevant, as higher adherence to the MeDi has been consistently associated with improved cardiometabolic outcomes, including reduced risk of CVD, improved metabolic control, and lower overall mortality as shown in a systematic review and meta-analysis by Furbatto et al. [52].

Notably, the increase in olive oil and fruit consumption observed in our cohort may have contributed to improved lipid profiles and metabolic parameters, as these components are known to enhance endothelial function and reduce cardiovascular risk, primarily through their content of monounsaturated fatty acids, polyphenols, and antioxidants [51]. A systematic review and meta-analysis of 30 randomized controlled trials demonstrated that olive oil consumption significantly reduces inflammatory markers (C-reactive protein and interleukin-6) and improves endothelial function, as measured by flow-mediated dilatation [53].

Interestingly, despite improvements in certain dietary components, a decrease in cereal consumption was observed. This finding warrants careful interpretation, as the MeDi emphasizes the intake of whole grains as a key source of dietary fiber and metabolic regulation [54,55].

Furthermore, the observed decrease in dairy products may have mixed implications. While excessive intake of certain dairy products has been debated, moderate consumption, particularly of fermented dairy, has been associated with beneficial effects on gut microbiota and metabolic health [56,57]. Therefore, the reduction observed in this study may not necessarily confer additional benefit and highlights the complexity of dietary modifications in cardiometabolic populations.

Despite these positive dietary shifts, the relatively low proportion of participants achieving high adherence underscores a critical gap between dietary recommendations and actual patient behavior. Recent studies have emphasized that even moderate adherence to the MeDi can confer health benefits; however, maximal protective effects are observed only at higher adherence levels [58]. The clinical relevance of these improvements should be interpreted in light of the dose–response nature of the relationship between MeDi adherence and health outcomes. Rather than conferring benefit only above a fixed threshold, adherence to the MeDi is associated with health outcomes in a largely linear, dose-dependent manner. A dose–response meta-analysis of 29 prospective cohorts reported that each 2-point increment in MeDi adherence was associated with an approximately 10% reduction in all-cause mortality, with a linear inverse association and no evidence of a threshold effect [59]. This dose-dependence is particularly relevant to the present population: an updated dose–response meta-analysis of nearly one million participants found that each 2-point increment in MeDi adherence was associated with an 8% lower risk of T2DM (HR 0.92; 95% CI 0.90–0.94), again following a continuous curve [60]. Accordingly, the significant increase in the continuous MDSS observed in the present population and the median score of 12 reached in the dietitian-followed subgroup likely reflects a clinically meaningful improvement in dietary quality, even in the absence of formally defined “high adherence.” The specific dietary shifts driving this improvement (greater intake of olive oil, fruit, and legumes, and lower red meat consumption) correspond to the core protective components of the MeDi. Nevertheless, because the greatest benefits are observed at higher adherence levels, the fact that most participants remained below the adherence threshold indicates meaningful room for further improvement and reinforces the rationale for sustained, individualized dietitian-led support.

### 4.4. Impact of Continuous Dietitian Led Follow-Up

The results of this study demonstrate that dietitian-led follow-up over a three-year period had a significant impact on the dietary habits of participants with T2DM and AH. Although differences in body composition parameters between the two subgroups were not statistically significant, except for phase angle, the subgroup of participants who regularly attended dietitian-led sessions exhibited a significantly higher total MDSS and a greater proportion of individuals with high adherence to the MeDi. A key finding of this study is that the subgroup under dietitian supervision reported significantly higher consumption of cereals, olive oil, nuts, fruit, vegetables, dairy products, and legumes, alongside lower consumption of red meat, a dietary profile that fully corresponds to the MeDi.

This finding is consistent with the existing literature emphasizing the critical role of continuous monitoring and nutritional counselling in achieving and sustaining dietary behavior change in patients with chronic disease. Evidence-based counselling, particularly in a collaborative model involving registered dietitians, has been shown to be effective in the management of AH and T2DM [61]. Furthermore, nutritional counselling delivered by dietitians as primary intervention providers effectively improves participants’ dietary habits, potentially leading to better individual health outcomes [62]. Dietitian-led interventions have been shown to significantly improve glycemic control, dietary adherence, and self-management behaviors [63]. Importantly, Busanello et al. showed that continuous follow-up and repeated counseling sessions appear to be more effective than single educational interventions, particularly in achieving sustained behavioral change and long-term metabolic improvements [64]. It is noteworthy that in this study only 34.2% of participants regularly attended scheduled follow-up appointments, which is consistent with the well-documented phenomenon of dropout from nutritional monitoring. In clinical settings, it has been reported that 40–50% of patients who initiate nutritional counselling do not return for follow-up visits, representing a significant challenge for long-term adherence [65]. Patient adherence to dietary recommendations and regular attendance at follow-up visits are key determinants of intervention success. Individuals who consistently attend scheduled consultations and remain engaged in the follow-up process are more likely to achieve sustained lifestyle changes and better metabolic outcomes [66]. Furthermore, patient motivation and self-efficacy play a crucial role in maintaining adherence over time, with higher motivation associated with improved compliance and clinical outcomes [67].

The absence of statistically significant differences in other body composition parameters between subgroups may be explained by several factors. The three-year follow-up period, combined with the relatively small proportion of regularly monitored participants (34.2%), may be insufficient to detect differences in anthropometric measures. Furthermore, the initial education provided at study enrolment was identical for all participants, meaning that the control subgroup also had access to foundational nutritional knowledge. Nevertheless, the finding that dietary quality was significantly better in the monitored subgroup confirms the clinical value of continuous dietitian-led follow-up as a component of comprehensive cardiometabolic patient management.

Early identification allows for prompt implementation of targeted interventions, including patient education and lifestyle modification, which are key strategies in slowing disease progression and improving long-term clinical outcomes [68].

Furthermore, continuous involvement of a clinical nutritionist throughout the course of treatment may improve adherence to prescribed dietary and therapeutic recommendations, enhance patient engagement, and support long-term self-management in individuals with diabetes and other chronic diseases. Current evidence emphasizes that individualized and ongoing nutrition therapy, integrated within a multidisciplinary model of care, represents an essential component of effective diabetes management and long-term cardiometabolic risk reduction [66,69,70]. Such an integrated, multidisciplinary approach may therefore optimize overall disease management and contribute to delaying the progression of CKD.

### 4.5. Limitations

Several limitations of this study should be acknowledged. First, the relatively high attrition rate may have introduced survivorship and self-selection bias, as participants who completed follow-up may have differed systematically from those who were lost to follow-up. Second, dietary intake was self-reported and therefore subject to both recall and social desirability bias, whereby participants may have over-reported foods perceived as healthy and under-reported those perceived as less desirable. Furthermore, objective dietary biomarkers, such as urinary polyphenols or plasma fatty acid profiles, were not collected; therefore, adherence to the MeDi could not be corroborated using objective measures. Third, the study was conducted during the COVID-19 pandemic, a period characterized by substantial disruptions in healthcare delivery, physical activity patterns, dietary behaviours, and social functioning. Moreover, more than half of the participants experienced COVID-19 during the follow-up period, which may have influenced nutritional status, body composition, and clinical outcomes.

In addition, given the chronic nature of T2DM and AH, changes in pharmacological treatment were expected over time. However, information regarding dose adjustments of existing medications and the initiation of new therapies, including lipid-lowering, antihypertensive, and glucose-lowering agents, was not systematically collected. Consequently, changes in biochemical and clinical parameters may have been influenced by pharmacological treatment in addition to dietary and lifestyle factors. An additional limitation concerns the generalizability of the findings. Participants were recruited from a nephrology outpatient clinic, and a substantial proportion had CKD or renal risk factors. Therefore, the results may not be fully generalizable to the broader population of individuals with T2DM and AH managed in other clinical settings.

Finally, allocation to the regularly followed and non-regularly followed groups was not randomized but resulted from self-selection, and participants who continued attending scheduled visits may have differed systematically from those who did not in motivation, health literacy, and general adherence to medical care. The observed association between dietitian-led follow-up and better dietary quality may therefore partly reflect a “healthy adherer” effect rather than an effect of the intervention itself. No multivariable adjustment for potential confounders was performed, and comparisons were unadjusted; the subgroup findings should accordingly be regarded as exploratory and hypothesis-generating. Moreover, multiple statistical comparisons were performed, increasing the possibility of type I error, and the observational design precludes the establishment of causal relationships between MeDi adherence and the observed clinical outcomes.

## 5. Conclusions

Over a three-year period, adults with T2DM and AH demonstrated a partial enhancement of MeDi adherence, accompanied by improvements in the lipid profile; however, in the absence of medication data the latter cannot be attributed to dietary change and may largely reflect pharmacological therapy. These changes were further accompanied by significant reductions in muscle mass and increased cardiovascular incidence. The present results thus emphasize that partial dietary improvement—and improvements in isolated biochemical parameters that may not be diet-driven—do not necessarily prevent progressive decline in muscle mass or rising cardiovascular burden. Continuous dietitian-led follow-up was associated with substantially greater dietary adherence and better nutritional indicators, yet a comprehensive approach remains warranted, one that integrates sustained dietary counselling, preservation of lean mass through adequate protein intake, structured resistance-based physical activity, and continuous renal and cardiovascular monitoring. As recommended by international diabetes, AH, and CKD guidelines, individualized and multidisciplinary management remains central to long-term risk reduction. By bridging the gap between controlled dietary interventions and real-world clinical outcomes, this study provides clinically relevant evidence to inform the integration of structured nutritional follow-up in the long-term management of cardiometabolic disease.

## Figures and Tables

**Table 1:** Comparison of baseline characteristics between participants included in the follow-up analysis and those lost to follow-up.

	Completed Follow-Up (*n* = 158)	Lost to Follow-Up (*n* = 90)	*p*-Value
Sex			
Male	95 (60.1)	48 (53.3)	0.30 *
Female	63 (39.9)	42 (46.7)	
Age (years), median (IQR)	65 (58–72)	71 (65–77)	<0.001 ^†^
Smoking	30 (19)	20 (22.2)	0.40 *
Chronic kidney disease	98 (62)	64 (71.1)	0.15 *
Malignant diseases	10 (6.3)	3 (3.3)	0.36 *
Cardiovascular diseases	12 (7.6)	15 (16.7)	0.02 *
Cerebrovascular diseases	2 (1.3)	5 (5.6)	0.10 *
Number of meals per day			
1–2	10 (6.3)	10 (11.1)	0.02 *
2–4	128 (81)	69 (76.7)	
>4	20 (12.7)	11 (12.2)	
MDSS, median (IQR)	8 (6–11)	8 (6–10)	0.55 ^†^
MDSS ≥ 14	9 (5.7)	11 (12.2)	0.07 *
E (×10^12^/L), median (IQR)	4.71 (4.33–5.11)	4.4 (3.9–4.7)	<0.001 ^†^
Hb (g/L), median (IQR)	137 (126–149.5)	129.5 (113.3–143.8)	0.001 ^†^
MCV(fL), median (IQR)	88.2 (85.3–91.7)	89.5 (86.9–93.2)	0.06 ^†^
Urea (mmol/L), median (IQR)	8.2 (6–12.2)	11.9 (8.4–16.5)	<0.001 ^†^
Creatinine (µmol/L), median (IQR)	112.5 (76–155)	148 (94.5–205.5)	0.001 ^†^
HbA1c (%), median (IQR)	6.9 (6.3–7.7)	6.9 (6.4–7.9)	0.88 ^†^
Glucose (mmol/L), median (IQR)	7.5 (6.5–8.8)	7.7 (6.5–9.5)	0.42 ^†^
Alb (g/L), median (IQR)	42 (39–45.5)	41 (38.1–44)	0.29 ^†^
Total cholesterol (mmol/L), median (IQR)	4.99 (4.2–5.8)	4.80 (4.1–6)	0.94 ^†^
LDL (mmol/L), median (IQR)	2.8 (2.2–3.5)	2.8 (1.8–3.7)	0.70 ^†^
HDL (mmol/L), median (IQR)	1.1 (0.9–1.3)	1.2 (0.9–1.4)	0.36 ^†^
Tgl (mmol/L), median (IQR)	2 (1.49–2.7)	1.9 (1.2–2.8)	0.33 ^†^
K (mmol/L), median (IQR)	4.5 (4.2–4.8)	4.5 (4.2–4.9)	0.94 ^†^
Ca (mmol/L), median (IQR)	2.37 (2.27–2.47)	2.40 (2.2–2.5)	0.72 ^†^
P (mmol/L), median (IQR)	1.14 (1–1.27)	1.10 (1–1.3)	0.67 ^†^
ACR (mg/g), median (IQR)	3.07 (0.71–20.87)	4.3 (1.1–57)	0.63 ^†^
Proteinuria (mg/24 h), median (IQR)	338.5 (93.5–1095)	879 (195–2466)	0.02 ^†^
Albuminuria (mg/24 h), median (IQR)	112.5 (18.75–787.5)	394 (39.8–1817.8)	0.06 ^†^
eGFR (mL/min/1.73 m^2^), median (IQR)	55.4 (35.3–80.28)	35.5 (23.9–69)	<0.001 ^†^
Uric acid (µmol), median (IQR)	409 (348–477)	430 (379.5–471)	0.15 ^†^

Abbreviations: IQR—Interquartile Range; MDSS—Mediterranean Diet Serving Score; E—erythrocyte count; Hb—hemoglobin (g/L); MCV—mean corpuscular volume (fL); HbA1c—hemoglobin A1c (%); Alb—serum albumin (g/L); LDL—low-density lipoprotein cholesterol (mmol/L); HDL—high-density lipoprotein cholesterol (mmol/L); Tgl—triglycerides (mmol/L); K—potassium (mmol/L); Ca—calcium (mmol/L); P—phosphates (mmol/L); ACR—albumin to creatinine ratio (mg/g); eGFR—estimated glomerular filtration rate using CKD-EPI (mL/min/1.73 m^2^). Data are presented as *n* (%) or median (interquartile range); * Chi-square test; ^†^ Mann–Whitney U test.

**Table 2:** Follow-up differences in the study population.

	Number (%) of Participants		*p*-Value
	Baseline (*n* = 158)	Follow-Up (*n* = 158)	
Sex			
Male	95 (60.1)	95 (60.1)	-
Female	63 (39.9)	63 (39.9)	
BMI (kg/m^2^)			
<18.5	1 (0.6)	0	0.02 ^‡^
18.5–24.9	15 (9.5)	23 (14.6)	
25–29.9	55 (34.8)	56 (35.4)	
>30	87 (55.1)	79 (50)	
Smoking	30 (19)	28 (17.7)	>0.99 ^†^
Chronic kidney disease	98 (62)	112 (70.9)	>0.99 ^†^
Malignant diseases	10 (6.3)	14 (8.9)	>0.99 ^†^
Cardiovascular diseases	12 (7.6)	40 (25.3)	< 0.001 ^†^
Cerebrovascular diseases	2 (1.3)	3 (1.9)	>0.99 ^†^
Number of meals per day			
1–2	10 (6.3)	2 (1.3)	<0.001 ^†^
2–4	128 (81)	38 (24.1)	
>4	20 (12.7)	118 (74.6)	
Surgeries *	-	31 (19.6)	-
Number of hospitalizations *	-	64 (40.5)	-
Had COVID-19 *	-	83 (52.5)	-

Abbreviations: *n*—number; BMI—Body Mass Index (kg/m^2^); *p*-values were obtained with ^‡^ Marginal homogeneity test and ^†^ McNemar-Bowker test; * during the follow-up period.

## Data Availability

The original contributions presented in this study are included in the article/Supplementary Materials. Further inquiries can be directed to the corresponding author.

## Supplementary Materials

The following supporting information can be downloaded at: https://www.mdpi.com/article/10.3390/nu18152482/s1, Table S1: Follow-up differences in the anthropometric parameters and body composition of the study population; Table S2: Follow-up differences in the laboratory parameters of the study population; Table S3: Follow-up differences in anthropometric parameters and body composition according to dietitian follow-up.

## Abbreviations

The following abbreviations are used in this manuscript:

T2DM	Type 2 diabetes mellitus
AH	Arterial hypertension
CVD	Cardiovascular disease
CKD	Chronic kidney disease
MeDi	Mediterranean diet
MDSS	Mediterranean Diet Serving Score
BMI	Body mass index
LDL	Low-density lipoprotein
HDL	High-density lipoprotein
Tgl	Triglycerides
eGFR	Estimated glomerular filtration rate
BIA	Bioelectrical impedance analysis
PhA	Phase angle
SMI	Skeletal muscle index
WC	Waist circumference
HC	Hip circumference
MUAC	Mid-upper arm circumference
FBG	Fasting blood glucose
HbA1C	Glycated hemoglobin
CRP	C-reactive protein
K	Potassium
P	Phosphates
Ca	Calcium
Alb	Serum albumin
E	Erythrocyte count
MCV	Mean cellular volume
IQR	Interquartile range
