# Supplementary material for: Rethinking Mediterranean Diet Adherence and Body Composition: A 3-Year Prospective Observational Study in Patients with Type 2 Diabetes and Arterial Hypertension
Source: Nutrients. 2026 Aug 1;18(15):2482. doi: 10.3390/nu18152482 (PMC13468672; doi:10.3390/nu18152482)
Supplement: Supplementary file 1 [file nutrients-18-02482-s001.zip › nutrients-4414392-supplementary.pdf]

Table S1. Follow-up differences in the anthropometric parameters and body composition of the study population

	Median (IQR)		
	**Baseline**	**Follow-up**	***p****
Age (years), median (IQR)	65 (58 - 72)	68 (62 - 74)	<0,001
**ANTHROPOMETRIC PARAMETERS**			
Height (cm)	174 (168 - 180)	174 (168 - 180,25)	0,86
Weight (kg)	95,15 (81,58 - 108,63)	92 (79,1 - 104,43)	<0,001
BMI (kg/m^2)	30,9 (27,83 - 34,88)	29,9 (27 - 33,33)	<0,001
Middle upper arm circumference (cm)	32,6 (29,98 - 36)	30 (28 - 33,5)	<0,001
Waist circumference (cm)	111 (102,53 - 120)	104 (96 - 116)	<0,001
Hip circumference (cm)	113,5 (106,38 - 124,13)	108,25 (101 - 118,5)	<0,001
WHtR	0,64 (0,59 - 0,69)	0,6 (0,55 - 0,68)	<0,001
WHR	0,96 (0,9 - 1,02)	0,97 (0,91 - 1,03)	0,56
**BODY COMPOSITION**			
Body fat (%)	28,05 (20,55 - 37)	28,75 (23,98 - 35,68)	0,45
Body fat (kg)	29 (23,25 - 36,675)	27,35 (19,35 - 34,6)	0,01
Fat-free mass (kg)	65,85 (58,775 - 75,025)	63,9 (57,1 - 72,38)	<0,001
Trunk fat mass (kg)	14,4 (10,1- 19,43)	14,5 (10,7- 19,75)	0,59
Trunk fat mass (%)	28,5 (22,35- 34,03)	29,8 (22,65- 34,6)	0,02
Muscle mass (kg)	62,55 (55,78 - 71,23)	60,7 (54,2 - 68,78)	<0,001
SMI (Skeletal muscle index)	9,08 (8,19- 10)	8,91 (7,85- 9,74)	<0,001
Phase angle (°)	5,7 (5,08 - 6,3)	5,1 (4,5 - 5,7)	<0,001

Abbreviations: IQR- Interquartile Range; BMI—Body Mass Index; WHtR—waist to height ratio; WHR— waist to hip ratio; **p*-values were obtained with the Wilcoxon test.

Table S2. Follow-up differences in the laboratory parameters of the study population

	Median (IQR)		
	**Baseline**	**Follow-up**	***p****
E	4,71 (4,33 - 5,11)	4,69 (4,35 - 5,05)	0,15
Hb (g/L)	137 (126 - 149,5)	138 (125,75 - 146)	0,39
MCV (fL)	88,2 (85,25 - 91,675)	89,95 (86,23 - 93,4)	**0,06**
Urea (mmol/L)	8,2 (6 - 12,2)	9,9 (6,4 - 15,1)	**<0,001**
Creatinine (mmol/L)	112,5 (76 - 155)	120,5 (77,75 - 184,5)	**<0,001**
HbA1c (%)	6,9 (6,3 - 7,7)	6,8 (6,3 - 7,5)	0,28
Glucose (mmol/L)	7,5 (6,5 - 8,8)	7 (6,15 - 9,05)	0,49
Alb (g/L)	42 (39 - 45,5)	42,3 (39,58 - 46,38)	0,65
Total cholesterol (mmol/L)	4,99 (4,2 - 5,8)	4,3 (3,55 - 5,15)	**0,002**
LDL (mmol/L)	2,8 (2,2 - 3,5)	2,3 (1,7 - 3)	**0,002**
HDL (mmol/L)	1,1 (0,9 - 1,3)	1,2 (1 - 1,5)	**0,001**
Tgl (mmol/L)	2 (1,49 - 2,7)	1,7 (1,3 - 2,5)	0,08
K (mmol/L)	4,5 (4,2 - 4,8)	4,6 (4,2 - 4,9)	0,88
Ca (mmol/L)	2,37 (2,27 - 2,47)	2,38 (2,28 - 2,47)	0,42
P (mmol/L)	1,14 (1 - 1,27)	1,24 (1,07 - 1,42)	0,04
ACR (mg/g)	3,07 (0,71 - 20,87)	3,92 (1,22 - 50,72)	>0,99
Proteinuria (mg/24 h)	338,5 (93,5 - 1095)	300 (122,75 - 946,25)	0,91
Albuminuria (mg/24 h)	112,5 (18,75 - 787,5)	88,5 (17,25 - 491)	0,65
eGFR (mL/min/1.73m^2)	55,4 (35,3 - 80,28)	44,7 (28,9 - 78,9)	**0,001**
Uric acid (mmol/L)	409 (348 - 477)	360 (303 - 414)	**<0,001**

Abbreviations: IQR- Interquartile Range; E—erythrocyte count; Hb—hemoglobin (g/L); MCV—mean cellular volume (fL); HbA1c— hemoglobin A1c (%); Alb—serum albumin (g/L); LDL—low-density lipoprotein cholesterol (mmol/L); HDL—high-density lipoprotein cholesterol (mmol/L); Tgl—triglycerides (mmol/L); K—potassium (mmol/l); Ca—calcium (mmol/L): P—phosphates (mmol/L); ACR—albumin to creatinine ratio (mg/g); eGFR—estimated glomerular filtration rate using CKD-EPI (ml/min/1,73m^2); **p*-values were obtained with the Wilcoxon test.

Table S3. Follow- up differences in anthropometric parameters and body composition according to dietitian follow-up

	Median (IQR)			
	No Dietitian follow-up (n = 104)	Dietitian follow-up (n = 54)	Total (n=158)	*p**
**ANTHROPOMETRIC PARAMETERS**				
Height (cm)	174 (169 - 180)	173 (166,75 - 181,5)	174 (168 - 180,25)	0,71
Weight (kg)	90,95 (78,9 - 102,73)	95,35 (81,58 - 109,03)	92 (79,1 - 104,43)	0,28
BMI (kg/m^2)	29,9 (26,93 - 32,68)	30,1 (27,45 - 35,53)	29,9 (27 - 33,33)	0,29
Middle upper arm circumference (cm)	30 (27,25 - 33)	31 (28 - 34)	30 (28 - 33,5)	0,39
Waist circumference (cm)	104 (96,25 - 113,38)	104 (95,75 - 117,25)	104 (96 - 116)	0,82
Hip circumference (cm)	107 (100 - 118)	111 (103,88 - 120,13)	108,25 (101 - 118,5)	0,19
WhtR	0,6 (0,55 - 0,66)	0,61 (0,54 - 0,69)	0,6 (0,55 - 0,68)	0,98
WHR	0,97 (0,92 - 1,03)	0,95 (0,9 - 1,02)	0,97 (0,91 - 1,03)	0,15
**BODY COMPOSITION**				
Body fat (%)	28,1 (23,55 - 33,775)	29,9 (24,65 - 38,2)	28,75 (23,98 - 35,68)	0,20
Body fat (kg)	26,45 (18,5 - 32,975)	28,9 (21,5 - 41,08)	27,35 (19,35 - 34,6)	0,21
Fat free mass (kg)	64,2 (57,1 - 71,43)	63,05 (57,08 - 74,4)	63,9 (57,1 - 72,38)	0,98
Trunk fat mass (kg)	14,15 (10,48- 18,8)	15,9 (10,85- 21,65)	14,5 (10,7- 19,75)	0,25
Trunk fat mass (%)	29,8 (22,58- 33,9)	29,5 (23,5- 37,8)	29,8 (22,65- 34,6)	0,29
Muscle mass (kg)	61 (54,2 - 67,93)	59,9 (54,18 - 70,78)	60,7 (54,2 - 68,78)	0,98
SMI (Skeletal muscle index)	8,92 (7,85 - 9,79)	8,84 (7,84 - 9,7)	8,91 (7,85 - 9,74)	0,96
Phase angle (°)	5,0 (4,4 - 5,68)	5,3 (4,8 - 5,8)	5,1 (4,5 - 5,7)	0,04

Abbreviations: IQR- Interquartile Range; BMI—Body Mass Index; WHtR—waist to height ratio; WHR— waist to hip ratio; *p-values were obtained with Mann Whitney U test.
